# PD42 Should Antibiotics Be Used To Treat Recurrent Otitis Media In Children? Updating A Recommendation

**DOI:** 10.1017/S0266462324003052

**Published:** 2025-01-07

**Authors:** Alba Ruiz-Ramos, Trinidad Sabalete-Moya, Beatriz Carmona-Hidalgo, María Piedad Rosario Lozano, Rocío Fernández-Urrusuno, Juan Antonio Blasco-Amaro

## Abstract

**Introduction:**

Acute otitis media (AOM) is one of the most common childhood infections. Recurrent AOM (rAOM) is defined as the presence of three or more AOM episodes in a period of six months. We describe the methodology used to update the recommendation of the 2018 Spanish National Antimicrobial Therapeutic Guide on the use of antibiotic treatments for rAOM in children.

**Methods:**

We followed the GRADE-ADOLOPMENT approach to update the recommendation on antibiotic treatment for rAOM. Firstly, the research question was framed in a Population, Intervention, Comparison, and Outcome format. A comprehensive search strategy was developed, the results of which were screened according to the inclusion criteria. The selected studies were reviewed, and the quality of the evidence was assessed. Subsequently, an Evidence to Decision (EtD) framework was created and the new evidence was presented to the Guideline Development Group (GDG), which updated the recommendation on rAOM treatment in children.

**Results:**

Among the 1,934 references identified by the database searches, only one guideline from the National Institute for Health and Care Excellence (NICE91, updated in 2022) met our inclusion criteria. This CPG included five individual studies comparing antibiotic treatments for rAOM. None of the studies demonstrated a significant advantage for any treatment. The overall quality of the evidence for these comparisons was considered low. A GRADE EtD framework was elaborated using the NICE91 recommendations but contextualized to the Spanish National Health System. Based on the evidence, the GDG did not modify the current recommendation provided in 2018.

**Conclusions:**

The overall quality of the available evidence regarding antibiotic use for rAOM in children was considered low. Further research is therefore needed to resolve the controversy and increase confidence in the appropriateness of using antibiotics in the treatment of rAOM, thereby improving the quality of life of children with this condition.

